# How Goes the Fight?

**Published:** 1921-03-05

**Authors:** 


### The Hospital

The Workers' Newspaper of Administrative Medicine and Institutional Life, Administration, National Insurance and Health.

No. 1813, Vol. LXIX. SATURDAY, MARCH 5, 1921. PRICE TWOPENCE

### HOW GOES THE FIGHT?

Generally speaking there is a simple technical reason explaining the early appearance of the accounts of provincial hospitals and the late publication of those belonging to the hospitals of London. In the Metropolis it is required that the income and expenditure statement shall relate to the exact year; necessarily, until the tradesmen's bills for December have been settled, a hospital cannot complete its accounts. In the provinces often cash payments only are taken, although usually they relate to the period December 1 to November 30.

Again, in London, the Central Funds, which are often the largest individual supporters of the hospitals, do not require the accounts very early, and their issue is not made until the governors' meeting, which is fixed, as a rule, as late as the by-laws of a hospital permit. Another factor, which illustrates the most important feature which differs as between London and the provinces, is that the provincial hospital most often serves a definite district, which is keenly interested in its local hospital and likes to see the accounts at the earliest possible moment.

The days, then, are early to obtain a satisfactory impression of the result of the fight to balance income and expenditure in which the voluntary hospitals were engaged in 1920; this will not be decided until, to quote the manifesto of the King's Fund, the uncertainties of the future of the voluntary hospitals are removed, and, the public knowing the true position, a better response to appeals for voluntary contributions is forthcoming. So we must wait for the report of Lord Cave's Committee before we can see clearly into the future. As to finances—and the difficulties of the hospitals are not entirely financial—we are able to glean some information as the days go by. The recent report of King Edward's Hospital Fund, containing the declaration of policy, tells us something of the financial position at the end of 1920, but as, notoriously, no accountant cares to issue approximate figures, it is possible that to be on the safe side most of the estimates of the finances of the Metropolitan hospitals were what are called conservative figures. To summarise the statement, it may be said that there was a deficit on 113 hospitals of £394,000 for the year, but this before the emergency grant of the King's Fund (£250,000) and the grants of the National Relief Fund (£200,000) to reduce debt had been taken into consideration. But of the 113 hospitals forty-two estimated that they actually would be able to show surpluses of income over expenditure, and we think there is reason to hope that the exact figures for the year will prove to be much more satisfactory than those estimated.

The annual reports are coming in, those from the provinces first and those of London following. As we expected, the position of the larger hospitals is less cheering than that of the smaller; for instance, a hospital of the class represented by the Manchester Royal Infirmary had a deficit of £28,577, while one of a smaller group, Sunderland Royal Infirmary, came off with a deficit of less than a thousand pounds. But the rule is not universal, as Glasgow Royal Infirmary, counting free legacies and large donations which by rule are not included in ordinary income, had a surplus. Speaking generally of provincial reports received to date, there is no depressing story of a hopeless financial position.

As to London, for the reasons stated, very little definite information is yet procurable. The annual meeting of King's College Hospital, however, affords some interesting facts and figures. For the first time in the history of the new building, the whole of the hospital, including the portion completed during the war, was occupied, with the exception of one ward, by civilian patients during a complete year. It will be remembered that during the war a large part of the hospital was constituted a military hospital under the Territorial scheme. The total expenditure for the year was £133,739, which is more than six times what it was in 1913, when, of course, the establishment was nothing approaching its present size. Although the hospital received grants of £17,500 from the British Red Cross Society through King Edward's Hospital Fund, and £22,200 from the National Relief Fund towards liquidating the war deficit, the excess of expenditure over income amounted approximately to £20,000. An interesting detail is that the cost per head on provisions has increased by less than 50 per cent.

as between 1914 and 1920. The revenue from patients' payments now amounts to £300 per week, and as this did not start until late in 1920 the hospital has something in the shape of increased income to look forward to.

At the Middlesex Hospital the Duke of Northumberland told the governors that they are " nothing like " making ends meet, and at the Prince of Wales's Hospital, Tottenham, Lord Gladstone announced that, in view of the financial outlook, the extension scheme has been reduced from a proposed expenditure of £102,000 to one of £44,860.

But at all the annual meetings that have yet been held there has been a remarkable absence of that note of despair which was so vibrant in the past difficult year. It is not our wish to discount in any way the findings of those who have larger and more accurate information before them, but if one can read anything from the signs of the times it is that the voluntary hospital system, in very much its present form, is likely long to outlive our day.

